# Impact of the Classic Chinese Garden Soundscape With Focus on Physiological and Psychological Effects, Tested Through Eye-Tracking, and Subjective Evaluation

**DOI:** 10.3389/fpsyg.2022.902630

**Published:** 2022-05-25

**Authors:** Minkai Sun, Lu Dong

**Affiliations:** ^1^School of Architecture and Urban Planning, Suzhou University of Science and Technology, Suzhou, China; ^2^College of Design, Jiaxing University, Jiaxing, China

**Keywords:** soundscapes, Chinese gardens, multi-sensory stimulation, Zhuozhengyuan, Tingyuxuan

## Abstract

Soundscape has been valued and practiced in classical Chinese garden designs. Some authentic patterns were even mentioned and used in gardening books hundreds of years ago. Though these patterns are well-known, how they work in a classic Chinese garden is still unclear. In this study, we chose one of the most famous soundscapes called Tingyuxuan (Listening to the Sound of Rain Hall) in Zhuozhengyuan (Humble Administrator’s Garden), Suzhou as the object. A video of the Tingyuxuan was captured on a rainy day, along with its sound. Twenty-four participants were asked to view this video twice (once with audio, once muted, in a random order). Eye-movement data and the subjective evaluation of participants were collected. The results showed that the participants’ visual attention is influenced by the sound of rain and helps them identify and observe the main element of the soundscape. Furthermore, participants experienced more positive feelings when viewing the video with the audio on.

## Introduction

In ancient China, soundscape has been recognized and explored in practice. Soundscapes were first constructed in the gardens of Zhou, Qin, and Han dynasties but these early forms of soundscapes in the environment were considered relatively simpler than today (Ao, 2020). It was only in the Ming Dynasty that there was a more detailed and integrated use of the sound in classical garden design (Ao, 2020). *Yuan Ye* is the only book on classical Chinese gardens and was written by Jicheng in the Ming Dynasty. He outlined the methods of using soundscapes to design classical gardens, such as “the rain falling on *Musa basjoo* at night,” “the breeze blowing through the willow branches,” and “the sound of the bluster of the wind” (Hongliu et al., 2019) in the book. He divided the natural sounds emanating from the garden into 13 categories, namely the sound of birds, insects, elk, dog barks, wind blowing through the plants, rain dropping on the plants, falling leaves, wind, rain, running water, waves, waterfalls, and spring water. He then put forward garden construction rules according to the soundscape in different environments (Hongliu et al., 2019; Ao, 2020). Ancient China adhered to the philosophy of “the unity of man and nature.” Sound, an important element in nature, has long been appreciated as being part of a specific kind of scenery (Hua et al., 2003; Yuan and Wu, 2008; Zhang and Liu, 2012). Zhuozhengyuan (The Humble Administrator’s Garden) was built by combining soundscape with garden plants, topography, and garden architecture (Guo, 2010; Ao, 2020). Soundscape can be regarded as an important part of classical Chinese gardens.

It is generally believed that modern soundscape research was proposed by Canadian musician R. Murray Schafer in the 1960s and has become one of the important directions for modern environmental research in general. The current research on soundscapes in gardens mainly includes four aspects: objective soundscape conditions, human feedback on soundscapes, how to establish soundscape databases and maps, and using soundscape elements for garden design (Wu, 2015). Most research on soundscapes applied a mixed-method, using a combination of objective measured data in sound level and subjective questionnaires. It involves the evaluation and analysis of people’s favorability and coordination of each element in the soundscape, as well as the study of emotion perception (Li et al., 2019; Xu and Liu, 2020). Wu (2015) proposed in his study of garden soundscapes some new methods and technologies, such as the use of three-dimensional integrated virtual technology in the laboratory to study the subjective and objective factors in the interactions between soundscapes and people.

The sound quality in cultural landscapes and natural spaces have been shown to have an impact on the viewer, especially as the human preference for natural sounds enhances mood satisfaction (Carles et al., 1999). For example, the influence of soundscapes in modern parks has been proven (Cao and Hsu, 2021; Jo and Jeon, 2021). The relationship between human behavior and a good environment can be determined by evaluating sound quality. In urban parks, even artificial sounds that destroy the original tranquil atmosphere can make people feel that the park is full of vitality (Elsadek et al., 2019). Adding natural sounds, like the singing of birds and the sound of water, in a noisy environment can reduce the traffic loudness and enhance sound quality (Hong et al., 2020; Jo and Jeon, 2020). Moreover, the preferred signal-to-noise ratio of bird song is significantly higher than that of water sound (Chitra et al., 2020). Moreover, in a study of soundscape perception in various urban parks, it was found that the effect of landscape effects on overall soundscape preference was more dependent on the preference for individual sounds (Fowler, 2010). By using multiple logarithmic models to explore the potential influencing factors of residents’ preference for urban gardens, it is found that the changes in the soundscape are closely related to the characteristics of the garden area. Residents like natural sounds, and traffic noise is the least favorite type of sound (Hong et al., 2021). In addition, regarding landscape differences, residents generally prefer natural urban gardens, without an artificial transformation and that prioritize the maintenance of natural landscapes and the historical and cultural heritage of urban gardens (Zhao et al., 2020).

The soundscape in modern landscape has a significant impact on human subjectivity and physiology. In terms of subjective evaluation, a questionnaire survey of 146 park users in West Lake Park, Fuzhou, China found that there is a positive relationship between the perception of the soundscape of urban parks and public visits. The soundscape’s tranquility shows the harmony of individual sounds, the perception of traffic sounds shows the most negative impact, while the perception of music-related and water sounds show a positive impact (Liu et al., 2019). People have negative emotions toward mechanical and traffic related sounds, and especially have significant negative effects due to dogs barking. Rhythmic sounds like music may be the only ones that actively contribute to satisfaction (Liu et al., 2019). Human activities are also affected by sound levels (Cristina, 2014). Under the same sound source, the landscape environment and topography will greatly affect people’s satisfaction (Fan et al., 2021). In the sound environment of urban parks, unpleasant traffic noise can be eliminated by introducing first choices such as bird chirping and water sounds. Dense vegetation, especially trees, can regulate the environment. Therefore, the volume, pleasure, and annoyance of the soundscape can be manipulated by introducing landscape elements (Chitra et al., 2020). When formulating policies and design measures, it is essential to consider the value that a soundscape adds to users and their activities. A soundscape evaluation questionnaire can be used and words such as “worries” and “pleasure” can be used to describe urban residents’ experiences. Based on the results of this open questionnaire, the tools used by local, regional, and national authorities can be adjusted (Bild et al., 2018).

In the study of soundscape effects on human physiology, positive changes have been reported in objective data, such as heart rate, respiration rate, and forehead electromyography (Hume and Ahtamad, 2013). Moreover, when people heard unpleasant sound segments, their heart rate dropped, or also, the more pleasant the sound, the greater the increase in breathing rate. The results of the forehead electromyography display that those unpleasant sounds have a significant effect on both men and women (Hume and Ahtamad, 2013). During recovery from stress and when resting, the function of the autonomic nerve may be affected by the subjective response to the acoustic environment. Differences between subjective estimates of the soundscape and physiological responses allow the establishment of corresponding scores to predict overall health and well-being outcomes that are independent of subjective evaluation (Medvedev et al., 2015). The difference between the subjective estimation of the soundscape and the physiological response can be taken as a kind of public consideration (Medvedev et al., 2015). Current research demonstrates that soundscapes are of great significance in the construction of park environments and have direct physiological effects on the human body (Liu et al., 2013). However, there is no specific research on what effect soundscapes can have on people in Chinese classical gardens.

Japanese gardens have their origins in China and have since been developing their own unique style and esthetic sense, with an emphasis on the use of natural materials to provide meditation spaces. The use of natural sounds as a fundamental design element has proven to have a close relationship with visual appreciation when designing Japanese gardens (Cerwén, 2019). In the Yugawara Garden of Tokyo, the physical expression of the landscape was balanced through the integration of terrain, planning, path structure, and visual contact, as well as the acoustic behavior related to these elements (Cerwén, 2020). Further, the link between soundscape and visual appreciation has been of interest in research (McConkie and Zola, 1979; De Lucio et al., 1996; Kohlrausch and Van de Par, 2005; Covic et al., 2017; Merlino et al., 2022), whereby images and sound have a reciprocal role in the perception of the overall quality of the landscape (Carles et al., 1999). Soundscape enhances the experience in a garden. Many classical gardens have special soundscape attractions, such as the wind blowing through the pine forest. In a soundscape study of nine classical gardens in Suzhou that are listed as World Heritage Sites (including the Humble Administrator’s Garden, which served as the location for this experiment), it was found that only 32% of tourists paid attention to the sounds in a garden, and 42% of them did not even notice. The soundscape in the gardens is overpowered by noise, although said noise is not very loud (Zhao, 2017). In a study on the auditory experience of a Japanese garden, it was found that the horticultural esthetics of the soundscape in the Japanese garden can be used as a model for urban sound design and architecture, which has long-term research significance. The combination of soundscape and vision can create balance and a sense of harmony in the garden (Fowler, 2010, 2013).

Between 1636 AD and 1912 AD, the design of soundscapes in classical Chinese gardens was well-established. Natural soundscapes accounted for about 90% of the total number of scenic spots, in which water scenes were the main type of natural soundscape. Often, gardeners would borrow elements such as plants and buildings to accentuate the sound in order to make it more easily perceived (Yang et al., 2016; Zhang, 2017). Japanese gardens have been influenced by Chinese gardens and, within the last few years, research has been proposed to develop the application and potential of sound action as a design tool for traditional Japanese parks, by combining sensory impressions, spatial relationships, and active participation in the environment (Cerwén, 2020). Based on the fact that research focused on soundscapes in classical Chinese gardens are insufficient, the human impact of soundscapes in traditional Chinese gardens can be further investigated, building on the existing research on soundscapes and multi-sensory experiences in Japanese gardens.

Since the psychological principles of classic soundscape in classical Chinese gardens are still unclear, the present study aims to reveal the effect of sound on visual characteristics and subjective evaluation during the landscape viewing process through cross-contrast experiments. The study aims to find which auditory elements in the construction of classical garden soundscapes have an impact on the viewer’s visual attention and subjective feelings. This research plays an important role in understanding the artistic conception and creation methods of classical Chinese gardens.

## Methods

### Experimental Soundscape

**FIGURE 1:**
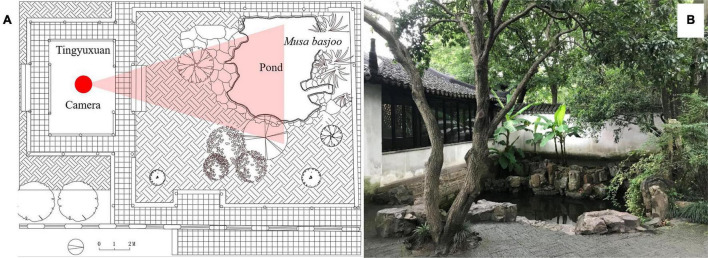
**(A)** The plan of Rain Hall [The red dots indicate the perspective at which the photograph **(B)** was taken]. **(B)** The image of the site (The video of the experiment at the later part is at the same perspective as the photograph).

To understand the role a soundscape plays in classic Chinese gardens, it was essential to use a flawless typical soundscape in the setting in this study. For this reason, we used the famous Tingyuxuan (Listening to the Sound of Rain Hall [Rain Hall]) in Zhuozhengyuan in Suzhou, China. Zhuozhengyuan has a 500-year history, and this garden is considered by some to be one of the finest gardens in southern China, in fact, it is one of UNESCO’s World Heritage Sites. The Rain Hall is in one corner of the garden, surrounded by 2 m-high walls. Thus, this scenery is relatively isolated from other parts of the garden. The isolation ensures that this portion is not influenced by other spots of the garden. Since Chinese classic gardens usually overlap multiple scene spot to enhance their performance, this place is suitable for the present study. Based on a classification of 62 soundscape sites in traditional Chinese gardens, the site for this experimental video was designed to emphasize the sound of rain through *Musa basjoo* plant and water ponds when the garden was built (Yang et al., 2016). The plan of Rain Hall is shown in Figure 1; the main element is a pavilion and the main view point is inside it. An atrium is spread in front the pavilion and beyond the atrium is a pond. *Musa basjoo* is planted in a corner of the atrium, beside the pond, and it is the main plant in this spot (Figure 1). The soundscape features two main elements, namely the *Musa basjoo* plant and water. The *Musa basjoo* is a broad-leafed plant, which makes a certain type of sound when raindrops fall on its leaves. The sound it emanates has been appreciated by Chinese scholars and artists for centuries (Brown et al., 2011; Hongliu et al., 2019; Hong et al., 2020). Specifically, the water sounds are mainly created by rain, since the pond is static.

### Experimental Setting

**FIGURE 2:**
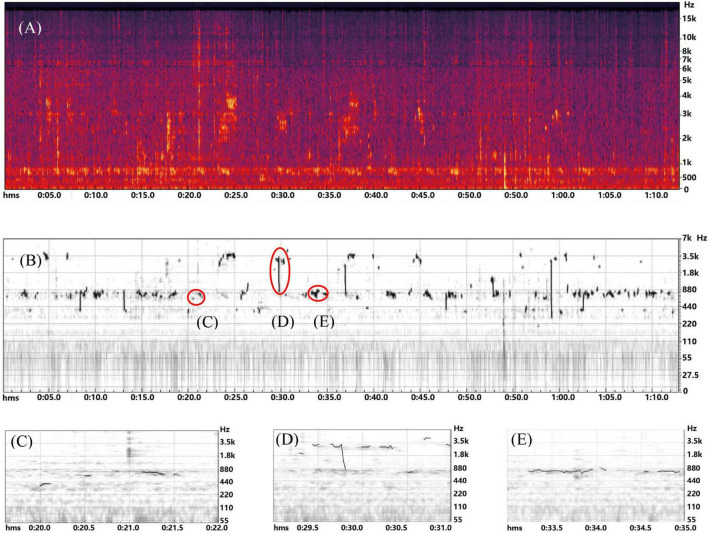
Panels **(A,B)** are the spectrograms and pitch analysis generated after noise reduction using Adobe Audition 2020 software. Panels **(C–E)** are the samples of pitch changes in the video when the rain beat the *Musa basjoo* and the tree leaves shacked simultaneously (20–22, 29–31, and 33–35 s).

A minute-long video was recorded by an iPad Pro camera (1920×1080 px, 60 fps/second, mono microphone). The video was taken on a moderately rainy day, with no wind and no visitor was around, at 7:00 a.m. The vantage point was inside the pavilion, with the camera facing the pond and *Musa basjoo*. There were no visitors around this place when the video was taken. The sound of the video was analyzed in terms of spectrum and pitch using Adobe Audition 2020, which is a digital audio workstation developed by Adobe Computer Software Company (Anna et al., 2011; Jing-wen and Min-xi, 2021). In order to reduce the interference of ambient sound, the sound of the video was firstly noise-reduced by removing background noise and hum at 50 Hz and below, thus the graphs in Figure 2A Spectrum and Figure 2B Pitch change was generated. Based on a comparison between the sound of the rain falling on the *Musa basjoo* leaves in the Supplementary Video and Figures 2A,B, it could be initially determined that the spectrum of the sound was mainly between 2 and 3 kHz and the pitch variation was mainly between 0.3 and 3 kHz (the continuous sound between 0 and 1 kHz in Figure 2A was inferred to be the sound of raindrops falling on the ground or other object surfaces. This acoustic feature was used to demonstrate that the sound of rain falling on the *Musa basjoo* leaves was clearly identifiable in the rain sound, but did not provide a refined analysis of the other rain sounds in the video). The three feature times at which the leaves shake after the rain falls on the *Musa basjoo* (20–22 s, 29–31 s, and 33–35 s) were matched using the software in Figure 2B, showed that the pitch changes corresponding to these three time points in Figures 2C–E were more pronounced than the pitch changes in the time before and after. This provides more evidence that the sound on the rain hitting the *Musa basjoo* leaf had identifiable characteristics in a medium rain environment (Figure 2).

**FIGURE 3:**
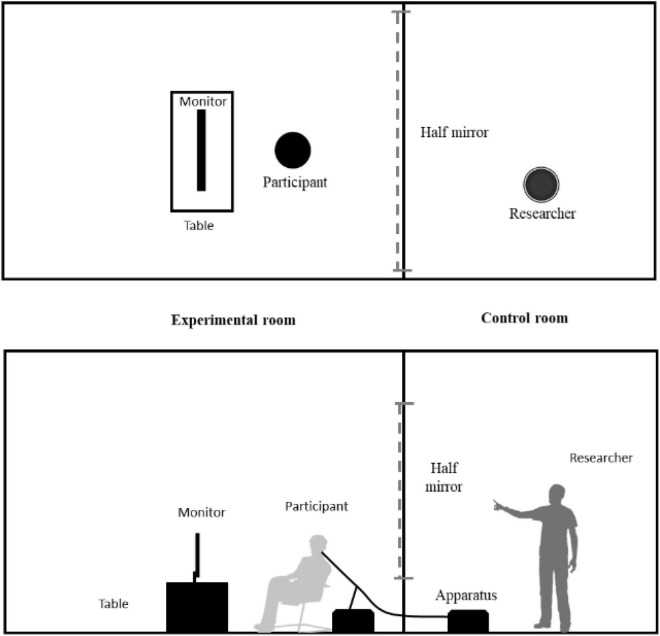
Experimental room setting.

The video was then replayed in a Windows computer with a 1920×1080 px, 16:9, 22-inch monitor. A two-speaker system was used (Philips, SPA311/93, 2-Channel, frequency response: 100 Hz–18 KHz, Signal Noise Ratio: 75 dB, China), with the speakers placed on either side of the monitor to simulate a real experience in the experimental room (5 m long, 3 m wide, and 3.5 m high) (Figure 3), specifically, a professional acoustic laboratory. The overall environment of the room was sound-absorbing, with ventilation and lighting systems designed to be soundproof. A Sound test scanner was used to check the ambient sound before the test to keep it within 15–30 dB to ensure that the acoustic test was carried out properly and without any interference from other ambient sounds. In addition, the rain sound in the video was tuned to approximately 50 dB, measured by the UNI-T (UT352, Uni-Trend Technology, China) professional noise instrument during the whole experiment, so that the experimental soundscape was reproduced as correctly as possible. An air conditioner and humidifier were used to maintain the temperature within the range of 16–18°C which would keep the same temperature between experiment room and the outside, and humidity was set at 50–60%. Beside the experimental room, a control room was also present, from where the researchers monitored the participants; researchers could observe the experimental room through a one-way grade glass and communicate with the participants *via* microphone.

### Participants

Twenty-four students from the Jiaxing University (10 male and 14 female, 19–21 years old) voluntarily participated in the study. They were all physically healthy with good eyesight (naked eye or soft contact lens, more than 0.7) and normal hearing. The expectation of space, which is based on memory, is an important factor in soundscape perception. Therefore, only those who never visited the room and garden were included in this experiment. Thus, we could focus on the effect and interaction of audio-visual multisensory stimulation. Human research protocols for the study were approved by the Ethics Committee of Jiaxing University (LS2019-105). Using the statistical program G*Power 3.1, we estimated that a minimum sample size of 23 was necessary for a paired–*t*-test, given α = 0.05, two tails, effect size dz = 0.6, non-centrality parameter sigma σ = 2.94, critical *t* = 2.07. Our sample of 24 was sufficiently powered, with actual power being always greater than 0.8.

### Measurements

Eye tracking and eye fixation were recorded using an eye-mark recorder (Tobii wearable eye tracking system, Tobii Pro Glasses 2). This device was used to measure eye movement. McConkie and Zola (1979) says saccadic movements take 20–50 milliseconds, while fixations last for approximately 200 milliseconds when reading texts and longer when looking at images (McConkie and Zola, 1979). Moreover, previous studies focused on relationship between visual attention on scenery defined fixations as reaching approximately 200 milliseconds (Sun et al., 2018; Elsadek et al., 2019). Thus, eye fixation duration was identified as being of at least 200 milliseconds.

When viewing a scene, eye movements reflect the patterns of visual exploration (De Lucio et al., 1996). In previous landscape scene studies that combined environmental psychology and landscape design, free viewing was used as a normal operation for the participants without a goal-oriented mechanism (Nordh et al., 2013). In an experiment using an eye-tracking device to record eye movements, it was found that people had fewer number of fixations for natural landscapes than for urban landscapes. Within this, fewer number of fixations were recorded for plants with leaves than for plants without leaves (Franěk et al., 2019). The significant relationship between fixation duration and interest has been demonstrated in many studies (Baker and Loeb, 1973; Underwood and Foulsham, 2006). Another study mentioned, more fixations in the same observation time increase the observer’s capacity to recognize and memorize what is represented (Duchowski, 2007). As a particular indicator, fixations represent the amount of attention involved in focusing on a scene (Berto, 2014). Greater eye fixation is thought to reflect the scene being viewed with more effort (Valtchanov and Ellard, 2015).

For the assessment of their overall subjective feelings associated with the video (including both sound and vision), the subjects were asked to fill out a questionnaire using a semantic differential (SD) method (Sadaaki and Masao, 1961; Guo, 2010). Based on previous studies (Sun et al., 2018; Cai et al., 2019; Elsadek et al., 2019), 17 pairs of adjectives (“like—dislike,” “comfortable—uncomfortable,” “familiar—unfamiliar,” “happy—sad,” “relieved—unrelieved,” “calm—agitated,” “natural—artificial,” “monotonous—varied,” “light—dark,” “tidy—messy,” “warm—cold,” “static—active,” “relax—tense,” “classic—modern,” “harmonious—discordant,” “pleasant—unenjoyable,” and “unique—ordinary”) were used in the questionnaire. For each pair of adjectives, participants evaluated the video on the scale ranging from −2 to 2. For example, if the pair of adjectives is “warm—cold,” then −2 (very warm), −1 (a little warm), 0 (neither hot nor cold), 1 (a little cold), and 2 (very cold).

### Experiment Procedure

When the participants arrived on the morning of the experiment, they were fully informed about the objectives, procedures, and how the instruments would be used. After receiving a description of the experiment, the subjects signed an agreement to participate. The experiment was performed from September 15 to 27, 2019. The time of the experiment was determined based on the weather forecast, avoiding sunny days so that participants would not feel the gap between the video content and the weather outside. Participants were instructed to turn off their mobile phones. In the experiment room, the participant sat on a comfortable chair, in the most comfortable position for them, 50 cm away from the monitor. The eye tracker device was placed in front of the participant to accurately record eye movement. The calibration for eye movement was carried out. The participant was instructed to relax and close their eyes and not move their head. While the participant rested with their eyes closed, the first video was replayed on the monitor. Simultaneously, the examiner asked the participant to open their eyes. The eye movements were recorded for 60 s. The participant was then asked to close their eyes again. After completing the psycho-physiological measurements, the participant was asked to fill out the SD questionnaire for the recent video. Then the whole process was repeated using the same video without sound. The order of the video was randomized across the participants (video with sound [VS] and video muted [VM]) in order to avoid habituation effect. The total procedure lasted approximately 15 min for each participant.

### Data Analysis

**FIGURE 4:**
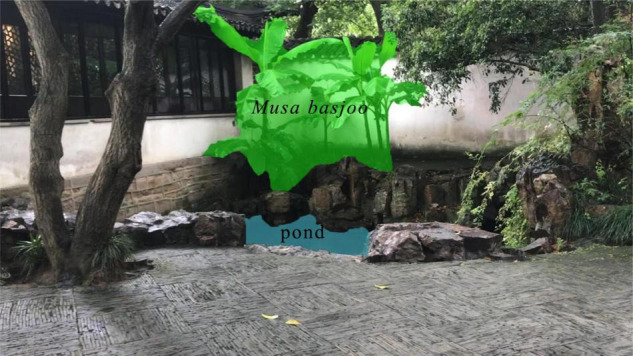
The two areas of interest (AOI) of the video scenery in this experiment are divided into ranges. Green area for the *Musa basjoo*, blue area for the pond, with the remaining part of the scene being background.

A paired *t*-test was used to verify the differences in psychological effect assessed by the SD scale. SD data were analyzed by comparing rating scores between the videos in the test. For the eye-movement data: to characterize the eye movements, two individual elements were identified—-*Musa basjoo* and Pond—-and the remaining part of the scene is background, completed the visual field, that is the two areas of interest (AOI) and the background in this experimental analysis (Figure 4). We first analyzed the number of eye fixation points, the duration of fixation at each point, and the average duration of eye fixation. Then, we calculated the ratio of the result of each element, juxtaposing it to the total number, and compared the ratio of the same element in two different videos. Then, all data were analyzed by the paired *t*-test. In all cases, the significance level was set at *P* < 0.01 and *p* < 0.05, respectively. All data are shown as mean ± SE (standard error) and 95% CI. All statistical analyses were performed using the Statistical Package for Social Sciences (SPSS) version 26.0 software (IBM Corporation, Chicago, IL, United States).

## Results

### Eye Movement

**FIGURE 5:**
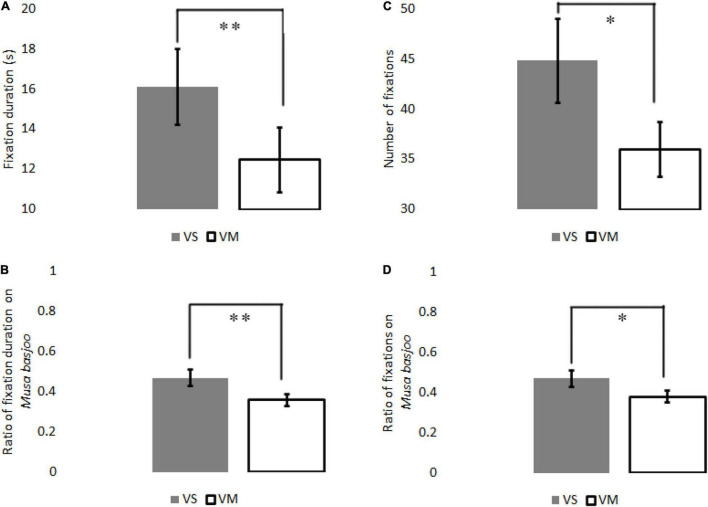
**(A)** The comparison of fixation duration between the two videos. **(B)** The comparison of ratio of fixation duration on *Musa basjoo* between the two videos. **(C)** The comparison of number of fixations between the two videos. **(D)** The comparison of ratio of number of fixations on *Musa basjoo* between the two videos. Data are shown as means ± SEM (*n* = 24, *p < 0.05, ^**^p < 0.01). VS, video with sound; VM, video muted.

The fixation duration of VS on *Musa basjoo* is significantly longer compared to that of VM, Cohen’s d (based on differences) with Hedges correction = 0.57. Moreover, the number of fixations of VS on *Musa basjoo* is significantly greater compared to that of VM, Cohen’s d (based on differences) with Hedges correction = 0.45. Ratio of fixation duration on *Musa basjoo* was significantly greater when viewing the VS compared to VM, Cohen’s d (based on differences) with Hedges correction = 0.48. Ratio of fixations on *Musa basjoo* was significantly greater when viewing the VS compared to VM, Cohen’s d (based on differences) with Hedges correction = 0.35. A significant difference was not observed for the total fixation number, fixation duration, and average fixation duration between the whole viewing of both videos (Table 1 and Figure 5).

**TABLE 1:** Visual perception data on *Musa basjoo*.

	VS		VM		t (23)	*p*

	**Mean value**	**95% CI**	**Mean value**	**95% CI**		
The fixation duration	16.11 ± 1.89	12.20; 20.02	12.46 ± 1.63	9.05; 15.89	2.83	<0.01
The number of fixations	44.79 ± 4.15	36.22; 53.37	35.96 ± 2.72	30.33; 41.59	2.23	<0.05
Ratio of fixation duration	0.47 ± 0.04	0.35; 0.53	0.36 ± 0.03	0.29; 0.42	2.70	<0.01
Ratio of fixations	0.47 ± 0.04	0.37; 0.52	0.38 ± 0.03	0.33; 0.44	2.21	<0.05

### Subjective Evaluation

As Figure 5 shows, seven pairs of adjectives corresponded significantly to the types of videos. For each of them, the evaluations when viewing VS was significantly more positive than when viewing a VM. Participants felt more familiar when viewing VS compared to VM, Cohen’s d (based on differences) with Hedges correction = 0.65. Participants felt calmer when viewing VS compared to VM, Cohen’s d (based on differences) with Hedges correction = 0.57. Participants’ opinions were more varied when viewing VS compared to VM, Cohen’s d (based on differences) with Hedges correction = 0.60. Participants felt brighter when viewing VS compared to VM, Cohen’s d (based on differences) with Hedges correction = 0.46. Participants felt warmer when viewing VS compared to VM, Cohen’s d (based on differences) with Hedges correction = 0.67. Participants felt less static when viewing VS compared to VM, Cohen’s d (based on differences) with Hedges correction = 0.50. Participants felt a more pleasant sensation when viewing VS compared to VM, Cohen’s d (based on differences) with Hedges correction = 0.76 (Table 2).

**TABLE 2:** Subjective evaluation of the two videos.

	VS		VM		t (23)	*p*

	**Mean value**	**95% CI**	**Mean value**	**95% CI**		
Familiar	4.04 ± 0.17	3.68; 4.39	3.43 ± 0.18	3.06; 3.80	3.51	<0.01
Calm	4.61 ± 0.17	4.28; 4.95	3.75 ± 0.25	3.25; 4.25	−3.06	<0.01
Varied	3.46 ± 0.19	3.07; 3.85	2.82 ± 0.21	2.40; 3.24	−3.20	<0.01
Brighter	3.68 ± 0.20	3.27; 4.09	3.14 ± 0.19	2.75; 3.53	2.49	<0.05
Warm	3.14 ± 0.17	2.80; 3.49	2.57 ± 0.15	2.26; 2.88	3.62	<0.01
Static	2.79 ± 0.24	2.30; 3.27	3.54 ± 0.24	3.05; 4.02	−2.67	<0.05
Pleasant	4.36 ± 0.17	3.69; 4.38	3.00 ± 0.21	2.57; 3.42	4.07	<0.01

*Data are shown as means ± SEM (n = 24). VS, video with sound; VM, video muted.*

## Discussion

The results of present study showed that, when the VS video was played, the *Musa basjoo* area significantly gained more visual attention than the remaining parts of the whole scenic spot. This means that, in alignment with traditional theory, the *Musa basjoo* plays a main role in this classic soundscape. Though the two videos contained the same visual information, for instance, the movement of the leaves of *Musa basjoo* when rain fell on it, in the video with sound, centralized more visual attention, evidenced by more fixation counts and longer fixation duration, compared to a non-sound video. These phenomena were considered would appear when participants viewed something with greater interest, more carefully, and paying more attention (Baker and Loeb, 1973; De Lucio et al., 1996; Underwood and Foulsham, 2006; Duchowski, 2007; Berto, 2014; Valtchanov and Ellard, 2015). A previous study indicated that audio-visual synchrony could attract spatial attention (Covic et al., 2017). Moreover, soundscapes combined with visual contents can focus the visitors’ sensorial experience in a garden (Fowler, 2010). Besides, another study found that in Chinese protected areas, sound was verified as an important factor with visual perception (Xu and Wu, 2021). In the present study, the sound of rain worked as a navigation tool that led participants to pay more attention to the *Musa basjoo*. This result further confirmed previous studies’ findings are also true in a classical Chinese garden. Moreover, the sound of rain makes the entire scenic spot worthy of its name: listening to the Sound of Rain Hall, which relies on the sound of rain to attract visitors’ visual attention. The video captured the sound of rain falling on the pond surface, the ground as well as the plants. There was no significant difference in the visual content of the two types of videos. The only difference was the sound of raindrops falling on the *Musa basjoo*. As it is evident from the spectral analysis shown before, multiple sounds are present in the video, but the sound of raindrops on a *Musa basjoo* leaf blade represents a clear identifying feature. Perhaps, the sound of rain falling on the ground or the pond surface was too ordinary to arouse the participants’ attention, compared to that on the leaves.

Overall, the results of the subjective evaluation showed the scenic spot was more positively evaluated when the participants watched the video with audio. Previous studies indicated that, for the whole environment, water features could improve the acoustic and visual satisfaction (Jo and Jeon, 2021). Natural sounds can also reduce stress (Liu et al., 2018). In the present study, participants scored higher when evaluating the video with sound in the categories “pleasant” and “familiar,” compared to the video without sound, which is consistent with previous research. Besides, it strengthened the hypothesis that natural sound can improve subjective comfort. Participants felt more varied sensations and more activated when they watched the video with sound. The audio-visual stimulation, combining acoustic and visual stimulation, could provide participants with more factors to perceive. Interestingly, the participants felt calmer when viewing the video with the rain sound, compared to the video without sound. This result seems contradictory to their feeling related to “varied” and “active” adjectives. This phenomenon may be caused by the sound of rain dripping on the leaf: (1) previous studies showed, sound of running water has a relaxing effect (Hong et al., 2020), the rain drop sound may have similar effect; (2) as motioned early, the sound of rain drop led participants to pay more attention to the *Musa basjoo*, the activated eye- movement may cause the participants’ “active” feeling. Besides, when the participants watched the video without sound, subtle noises from computer fans and hard drives might have been more noticeable to them. Furthermore, in traditional Chinese literature, the saying “the more the cicadas scream, the quieter the forest” is widely known. The sound of rain in this study may have played a similar role to that of cicadas.

Present study focused on the effect and interaction of audio-visual multisensory stimuli of Chinese traditional garden soundscape. Specifically, other factors such as memory and knowledge of culture background was not discussed in present study. Stimulus is sometimes used to recall the participant’s memory for well-known soundscapes. Thus, for the site Tingyuxuan, people who know this place or have been there may react different from participants involved in present study. The effect of memory and knowledge of culture background should further be considered in future research.

Besides, as a preliminary exploration, the sound track of the video is mono, future studies should use binaural microphones or ambisonic microphone to reproduce the soundscape more realistically.

## Conclusion

The present study adopted an evidence-based approach to the experiment to explore how people’s visual attention and subjective evaluation were influenced by the typical Chinese traditional soundscape. One of the most significant findings in this study is the psychological and emotional effect that the sound of raindrops falling on leaves of *Musa basjoo* could have on people. Although the sound of raindrops on the pond surface and ground featured in the video, the sound from the leaves of *Musa basjoo* increased the participants’ fixation number and fixation duration. In addition, the participants’ subjective assessment scores were higher when the video contained rain sounds. The present study has provided insight into the design theory of environmental psychology in traditional Chinese soundscape. Findings in this study can help designers understand the relationship between audio and visual elements better and promote the revaluation of classic design techniques.

## Data Availability Statement

All relevant data is contained within the article: the original contributions presented in the study are included in the article and Supplementary Material, further inquiries can be directed to the corresponding author.

## Ethics Statement

The studies involving human participants were reviewed and approved by Ethics Committee of Jiaxing University (LS2019-105). The patients/participants provided their written informed consent to participate in this study.

## Author Contributions

MS managed the project, designed the task, conducted the human experiments, wrote the main manuscript text, and gave advice about the manuscript. LD managed the project, statistically analyzed the data, wrote parts of the main manuscript text, and prepared the figures and tables. Both authors have read and agreed to the final version of the manuscript.

## Conflict of Interest

The authors declare that the research was conducted in the absence of any commercial or financial relationships that could be construed as a potential conflict of interest.

## Publisher’s Note

All claims expressed in this article are solely those of the authors and do not necessarily represent those of their affiliated organizations, or those of the publisher, the editors and the reviewers. Any product that may be evaluated in this article, or claim that may be made by its manufacturer, is not guaranteed or endorsed by the publisher.
